# Overcoming challenges in the UK's National Health Service

**Published:** 2016

**Authors:** Andy Cassels-Brown, Darren Shickle, John Buchan

**Affiliations:** Leeds Ophthalmic Public Health Team, Academic Unit of Public Health, University of Leeds, UK.

**Figure:**
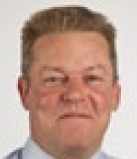
Andy Cassels-Brown

**Figure:**
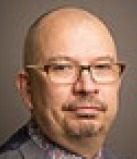
Darren Shickle

**Figure:**
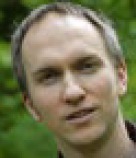
John Buchan

Working in an eye clinic in Dewsbury, West Yorkshire (with its large South Asian migrant population) in the 1990s, Andy Cassels-Brown noticed the large number of young South Asian patients who presented with much more advanced keratoconus than their Caucasian counterparts, who tended to be detected much earlier. This indicated an inequality in access to eye care services which, we discovered, was made worse as the Asian patients frequently had preventable associated allergic conditions (such as allergic conjunctivitis or eczema) and a strikingly strong family history of keratoconus.^1^ Better access to eye care would permit earlier identification of family members with the condition and, these days, prevention of progression by means of cross-linking to stabilise the keratoconic cornea.

After doing a Masters in Community Eye Health at ICEH London in 2000, Andy cycled daily to the eye department at St James's Hospital, through Leeds' multi-ethnic suburb of Chapeltown. Andy started to wonder why there were not more African-Caribbean patients with glaucoma coming to St James' Hospital, given the higher prevalence and earlier onset (but often all-too-late presentation) in this population group. Research confirmed^2^ that there were no optometry practices in this socio-economically deprived community, and that people found it difficult and expensive to come into the city centre for eye tests and to pay for prescription spectacles.

The Leeds Ophthalmic Public Health Team (which includes Darren Shickle, John Buchan and other colleagues) is part of the UK's National Health Service and is based at the University of Leeds. The team undertook a Glaucoma Health Equity Profile needs assessment across the whole city of Leeds, which confirmed that late presentation was highly linked with socio-economic deprivation.^3^ A VISION 2020 Equity Profile conducted across Leeds and Bradford^4^ also confirmed the links between high prevalence, late presentation, ethnicity and socio-economic deprivation. These studies were a clear demonstration of the inequalities present in the UK, despite its developed economy and world-renowned National Health Service (NHS), which offers universal access to health care.

Along the way, the team has tried to honour the well-known adage: ‘no survey without service’ and has undertaken health promotion campaigns for glaucoma, diabetes and smoking cessation in Leeds, including the use of community radio and health promotion stands at festivals and carnivals. We have also trained link workers in many locations across the city to talk to community groups, community support workers, social workers and staff members in elderly care homes about the importance of having regular sight tests.

As part of the VISION 2020 Leeds programme, we developed consultant-led multiprofessional Community Eye Centres. The centres are located in specific communities around Leeds. They help to target inequalities by offering eye care services to people who would otherwise face socio-economic and geographical barriers to accessing eye care.

The Leeds Ophthalmic Public Health Team have continued to research and pilot innovative ways to deliver primary eye care in areas of deprivation, including the provision of free sight tests and free prescription spectacles in the community.

**Figure:**
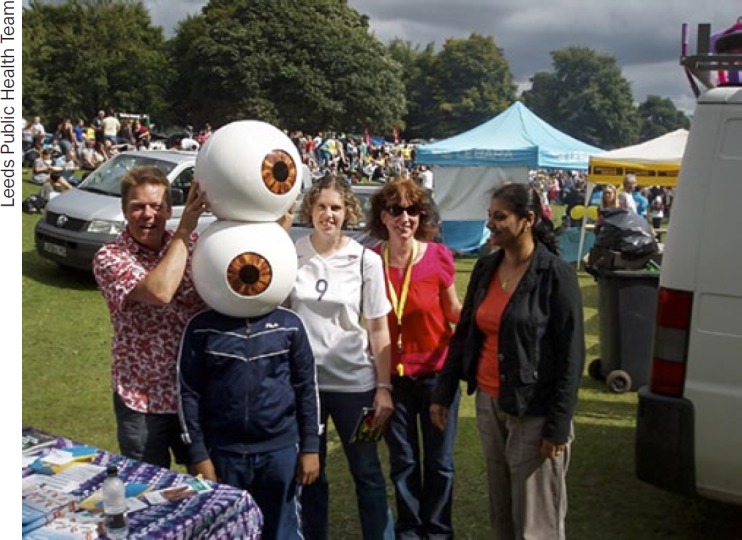
The Leeds Ophthalmic Public Health Team and students promoted eye health at Leeds Carnival. UK

We have identified that the number of people registered as blind in communities with high levels of deprivation and large ethnic populations is significantly lower than expected, considering what is known about the incidence of blindness in these communities. This suggests inequality in access to the registration system, which in turn excludes people from the state support offered to those registered as being visually impaired or blind.

In summary, despite our well-resourced National Health Service, there are many examples of inequality in access to services, and care often depends on where you live (known in the UK as the postcode lottery). Our biggest challenges now include increasing detection, prevention and curative service capacity to meet the increase in demand for hospital eye care in England (due to the ageing of the population and an increase in treatment options, e.g. for wet ARMD).

Our response in Leeds, as with much of the rest of the world, is to train our primary care workforce and continue to decentralise eye care by dealing with lower complexity cases in consultant-led, multiprofessional community settings, rather than in hospital. We are developing a multi-disciplinary academy to train ophthalmic nurses, optometrists, orthoptists, health care assistants and allied health professionals in line with an emerging ‘competency framework’^5^ which is currently being developed in the UK And finally, before it is too late, we are also starting to develop strategies to be resilient against the impact of environmental change and to reduce health care's damaging environmental footprint.
